# Public Notice

**Published:** 1864-03

**Authors:** 


### PUBLIC NOTICE.

Hereafter, the price of Hall's Journal of Health will be One Dollar and a half a year; single numbers Twelve Cents. Our readers will, we are sure, appreciate the necessity of this increase in charges without any further explanation.

All our publications — "Bronchitis," "Consumption," "Health and Disease," "Sleep," the two Bound Volumes of "The Fireside Monthly," (now discontinued,) and the ten Bound Volumes of "Hall's Journal of Health" — are at the uniform price of $1.25 each. By mail, post-paid, $1.50 each.

Home on the Hudson.—For sale, one of the finest Country Seats on the east bank of the Hudson, sixty-six miles from town, and commanding a beautiful river view. About forty acres, four square, every foot productive. A handsome double mansion, with all the modern improvements of furnace, gas, range, with hot and cold water in the chambers, bath-room, laundry, etc., porter's lodge, ice-house well filled, and commodious stables and carriage-house. All the buildings in the most complete repair. Over two thousand ornamental, shade, and fruit trees have been set out within the last ten years; large garden, with every variety of small fruit, etc. Unsurpassed for healthfulness. Ready for immediate occupancy. Carpets, mirrors, book-cases in library, gas chandeliers, etc., will go with the house. A large portion of the purchase money can remain for a term of years on bond and mortgage if desired. Reference is made to Dr. W. W. Hall, of New-York, Theodore B. Wetmore, Esq., of 31 Pine street, New-York, whose country seat adjoins the place; to Daniel Denny, Esq., President of the Hamilton Bank, Boston; Hon. Erastus Brooks, New-York, of Baltimore, Benj. T. Treedick, Esq., of Philadelphia, and Dr. Pliny Earle, at the United States Lunatic Hospital at Washington, D. C. The New-York Evening *Express* says of this valuable property that it is one of the most beautiful and healthy country seats upon the Hudson, complete in house, grounds, and stables, all properly furnished and fit to be occupied at once. Over two thousand fruit and ornamental trees are upon the place. The owner parts with this property only because he is about to leave the State.

Beautiful Hair.—"Dodge's Tincture," applied monthly, keeps the scalp free from dandruff, promotes, by its cleansing, agreeably stimulating properties, the growth of the hair where any other agent can, is an admirably soothing curative of wounds and sores of the scalp, and has now been discovered to destroy instantly, by one application, all vermin infesting the hair of children and domestic pets, and of persons sleeping in strange beds and otherwise. Small bottles, 25 cents; Quarts, $1.50. P. C. Godfrey, 831 Broadway, New-York.

Economy, Light, and Cookery—W. D. Russell, of 206 Pearl street, New-York, has patented a lamp attachment by which water is boiled and a comfortable meal prepared by a common lamp, which at the same time lights the room without interfering with the comfort of the person reading, writing, sewing, etc. Price 50 cents.

The "MEDICINE SHELF," published by the American Tract Society, 28 Cornhill, Boston, and by J. G. Broughton, 13 Bible House, New-York City, abounds in striking illustrations of useful and practical truths, in eighteen chapters, (315 pp. 16mo,) on Lifting the Curtain, My Neighbors, Square Corners, Sin and Sorrow, One Right Way, etc. Also, "Pictures and Lessons" for Little Readers, being ninety-six pictures and as many lessons, theoretical and practical, for young readers; every one of which will be literally devoured. "Sandy Maclean and two other Stories," all deeply interesting. Sargeant's Temperance Tales is well worthy of a place in every family, literally, whether temperate or intemperate. A most valuable present for any one to make to any one. "Home Stories for Boys and Girls," forty-seven stories. It is one of the most beautiful, interesting, and instructive volumes for children we have seen for many a day. Money can not be better laid out for family reading than by spending it at the New-York Bible House, number thirteen. We suppose the "other" Tract Society and the old-school Sunday-School Publishing House are reposing in their dignity, and are waiting for customers to come to them, for we seldom see any of their issues on our table, while the new-school branch are wide awake, and are making most of their time by letting the people know what delightful and useful reading they are preparing for them every week, for every week they have something new, and as good as it is new, and take pains to have them noticed in periodicals which go to families and households all over the land. Another of their interesting publications is "Our Father Who Art in Heaven," a story illustrative of the Lord's Prayer. Also, "Reposing in Jesus," or, the true secret of grace and strength, by G. W. Mylne, a book which every heart Christian can always feed upon and grow and thrive.

PHOTOGRAPHIC MAGNIFIER AND STEREOSCOPE COMBINED—sent by mail for $1.50—adds greatly to the beauty, interest, and value of all Stereoscopic and Photographic Pictures; it makes the Photographic Album more interesting, because it magnifies and brings out the features with such a life-likeness as to delight every one who takes up the instrument.

A NEW EDITION OF OUR BOOK ON SLEEP is just issued, treating of all subjects connected with the hours of sleep, and is of personal and practical application to all classes, ages, and conditions, especially to that multitude of youth who have been beguiled by advertisements in the daily papers under the headings of "Manhood," "Nervous Debility," "Physiology of Marriage," "Restored Powers," "Marriage Guide," etc. By mail, $1.50.

"HEALTH AND DISEASE" is entirely exhausted, but is promised by our printer to be ready by the tenth of March; $1.50 by mail. Our new book of "Two Hundred Health Tracts," with monograms on various interesting subjects, will appear at the same time. Price $1.75. By mail, post-paid, $2. Persons will be supplied in the order of their application.

